# Prescriptions of Chinese Herbal Medicines for Insomnia in Taiwan during 2002

**DOI:** 10.1093/ecam/nep018

**Published:** 2010-10-20

**Authors:** Fang-Pey Chen, Maw-Shiou Jong, Yu-Chun Chen, Yen-Ying Kung, Tzeng-Ji Chen, Fun-Jou Chen, Shinn-Jang Hwang

**Affiliations:** ^1^Center for Traditional Medicine, Taipei Veterans General Hospital, Taiwan; ^2^National Yang-Ming University School of Medicine, Taipei, Taiwan; ^3^Department of Family Medicine, Taipei Veterans General Hospital, Taipei 112, Taiwan; ^4^Graduate Institute of Integration Chinese and Western Medicine, Chinese Medical University, Taichung, Taiwan

## Abstract

Chinese herbal medicine (CHM) has been commonly used for treating insomnia in Asian countries for centuries. The aim of this study was to conduct a large-scale pharmaco-epidemiologic study and evaluate the frequency and patterns of CHM use in treating insomnia. We obtained the traditional Chinese medicine (TCM) outpatient claims from the National Health Insurance in Taiwan for the year 2002. Patients with insomnia were identified from the diagnostic code of International Classification of Disease among claimed visiting files. Corresponding prescription files were analyzed, and an association rule was applied to evaluate the co-prescription of CHM. Results showed that there were 16 134 subjects who visited TCM clinics for insomnia in Taiwan during 2002 and received a total of 29 801 CHM prescriptions. Subjects between 40 and 49 years of age comprised the largest number of those treated (25.3%). In addition, female subjects used CHMs for insomnia more frequently than male subjects (female : male = 1.94 : 1). There was an average of 4.8 items prescribed in the form of either an individual Chinese herb or formula in a single CHM prescription for insomnia. Shou-wu-teng (*Polygonum multiflorum*) was the most commonly prescribed single Chinese herb, while Suan-zao-ren-tang was the most commonly prescribed Chinese herbal formula. According to the association rule, the most commonly prescribed CHM drug combination was Suan-zao-ren-tang plus Long-dan-xie-gan-tang, while the most commonly prescribed triple drug combination was Suan-zao-ren-tang, *Albizia julibrissin*, and *P. multiflorum*. Nevertheless, further clinical trials are needed to evaluate the efficacy and safety of these CHMs for treating insomnia.

## 1. Introduction

Insomnia is a common health problem in the general population worldwide [1]. Individuals with insomnia may suffer from the inability to fall asleep, remain asleep, or have non-restorative sleep, thereby influencing their daytime functioning [2]. From an etiologic point of view, this sleep disorder can be characterized as primary insomnia, which does not result from physical or mental factors [3]. In contrast, secondary insomnia is caused by factors, such as psychological, psychosocial, or drug dependency [4]. However, occasionally there is no definite causal relationship between precipitating factors and the occurrence of insomnia; hence, this form of insomnia could be considered a co-morbid condition [5].

Therapy for insomnia in Western medicine is mainly based on prescribed medications such as benzodiazepines, antidepressants, anticonvulsants, or over-the-counter antihistamines [5]. However, these medications are sometimes associated with adverse effects and are not suitable for long-term use [5]. In addition to the aforementioned medications, there are behavioral and cognitive therapies or alternative and complementary treatments utilized in Western countries, such as melatonin, l-tryptophan, herbal passionflower, valerian and St John's wort [5, 6].

In Asian countries, traditional Chinese medicine (TCM) has been widely used for centuries [7–10]. Accordingly, Chinese herbal medicines (CHMs) are frequently used in the treatment of insomnia. Several studies have shown that treatment with CHMs effectively improve sleep quality, prolong sleep duration and exhibit fewer side effects than Western medicines, including lethargy, dry mouth and dizziness [11–13]. However, there have been no large-scale pharmaco-epidemiologic studies of CHMs for the treatment of insomnia. With respect to their use in clinical practice, prescriptions of CHMs for insomnia largely reflect the experience of the Chinese herbal doctor or what is recommended by traditional Chinese texts. Therefore, the optimal CHM prescription for the treatment of insomnia remains to be clinically or scientifically established.

In Taiwan, the National Health Insurance (NHI) program has reimbursed the medical expenses incurred for Western medicines for nearly all residents since 1995; NHI has provided for 22 520 776 beneficiaries as of the end of 2002, which includes nearly 97% of the total population in Taiwan [14]. The use of TCM has been reimbursed by the NHI since 1996, and the people of Taiwan are free to choose between practitioners of Western medicine or TCM. In addition, the Taiwanese are allowed to visit primary-care clinics or hospitals without a referral. Because all claim data are available to researchers in an electronic form, a large-scale survey of pharmaco-epidemiologic issues can be feasibly conducted. The aim of the current study was to explore the frequency and pattern of CHM use in subjects with insomnia by analyzing the NHI database for the year 2002 in Taiwan.

## 2. Subjects and Methods

### 2.1. Data Sources

The NHI program in Taiwan was implemented in 1995, and the NHI Bureau began to release all claims data in electronic form to the public under the National Health Insurance Research Database (NHIRD) project. The structure of the claim files is described in detail at the NHIRD Web site and in our previous publications [10]. In brief, we obtained the database of TCM claims from NHIRD, including the office-visit files and corresponding prescription files (CM_CD2002.DAT and CM_OO2002.DAT), for the year 2002 in Taiwan. The office-visit files recorded the dates of encounters, the medical-care facilities and specialties, the patient genders, the patient birth dates, and up to three diagnoses according to the International Classification of Diseases, Ninth Revision, Clinical Modification (ICD-9-CM). For privacy protection, the unique identifiers of the patients and institutions were scrambled cryptographically to assure anonymity. The prescription files contained the prescription records for the CHMs corresponding to the patient's office visits. A CHM prescription contained one or more Chinese herbs or herbal formulae. A single Chinese herb or herbal formula was processed into powder or fine granules in Taiwan, and were easily mixed and dispensed into small packages so that each prescription could be taken one at a time.

All TCM treatments covered by the NHI are provided only in ambulatory care clinics of Taiwan, and there is no inpatient care for TCM recipients. In addition, only licensed TCM physicians are eligible for reimbursement. The insurance benefits of TCM in Taiwan include CHMs, acupuncture and traumatologic manipulative therapy, which is especially designed for joint dislocations.

### 2.2. Study Design

Although the concept of disease states in TCM is quite different from that in Western medicine, the TCM physicians in Taiwan have been requested to code for office-visit claims with a diagnosis based on the ICD-9-CM designation (no more than three diagnostic codes at each visit). In this study, we chose the data of subjects with a single diagnostic code for insomnia (i.e., ICD-9-CM code 780.52) among the TCM visits.

Patient management via TCM often includes a single prescription from a TCM physician that may contain an individual Chinese herb or multiple herbs of various dosages. Examples include a compound (Fu-Fang), a classical formula (regimen, remedy or Fang-Ji) that is a combination of compatible Chinese herbs in fixed dosages according to classical or well-known texts of Chinese medicine, a classic formula combined with some Chinese herbs (Chia-Chien-Fang) or several formulae combined together with or without one or several Chinese herbs.

### 2.3. Data Analysis

The database software, IBM DB2 8.1, was used for data linkage analysis and processing. Regular statistics were displayed for the use frequency and patterns of CHM prescriptions for insomnia. Association rule mining, originally developed in the 1990s to identify which groups or sets of items were likely to be purchased together, was applied to analyze the prescription rates of the Chinese herbals for insomnia [15, 16]. The association rule was applied for the prescription analysis in the following manner: when a physician prescribed drug A (or drugs A1 and A2, drugs A1, A2 and A3, etc.), drug B is also prescribed in X% of cases, and this co-prescribing is present in Y% of all prescriptions. The support factor is the ratio of co-prescriptions of all prescriptions (i.e., Y% in the above example). The confidence factor is the ratio of co-prescriptions to prescriptions for drug A (i.e., X% in the above example). When executing the program to identify association rules in our data set, we chose 0.5% as the minimum support factor and 30% as the minimum confidence level [17].

## 3. Results

Among the 22 520 776 valid beneficiaries of the NHI at the end of 2002 in Taiwan, 6 221 426 subjects (27.6%) used TCM during that year and 16 134 subjects (0.3%) visited TCM clinics and exclusively used CHMs for insomnia. Among these subjects with insomnia, there were 29 801 CHM prescriptions. The peak age of these subjects with insomnia treated by TCM was between 40 and 49 years (25.3%), followed by 30–39 years (23.8%) and 50–59 years (17.0%; Table 1). In addition, female subjects used CHM for insomnia more frequently than male subjects (female : male = 1.94 : 1).

The most common individual Chinese herb prescribed for insomnia was *Polygonum multiflorum* (Shou-wu-teng; 23.8%), followed by *Ziziphus spinosa* (Suan-zao-ren; 18.3%), *Poria cocos* (Fu-shen; 13.3%), *Albizia julibrissin* (He-huan-pi; 10.0%), *Ostrea gigas* (Mu-li; 8.1%), *Polygala tenuifolia* (Yuan-zhi; 8.0%), *Saliva miltiorrhiza* (Dan-shen; 7.5%), *Scutellaria baicalensis* (Huang-qin; 7.4%), *Coptis chinensis* (Huang-lian; 5.4%) and *Lilium brownii* (Bai-he; 5.1%; Table 2).

Suan-zao-ren-tang (31.2%) was the most commonly prescribed Chinese herbal formula for subjects with insomnia, followed by Jia-wei-xiao-yao-san (21.2%), Tian-wang-bu-xin-dan (15.6%), Chai-hu-jia-long-gu-mu-li-tang (12.4%), Wen-dan-tang (11.6%), Gan-mai-da-zao-tang (11.5%), Gui-pi-tang (6.6%), Zhi-bai-di-huang-wan (6.0%), Long-dan-xie-gan-tang (4.9%) and Qing-xin-lian-zi-yin (4.6%; Table 3).

**Figure 1:**
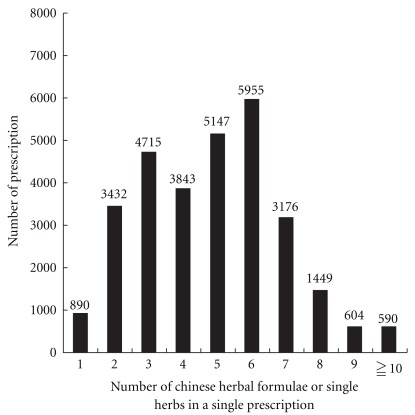
Relationship between the number of prescriptions in Taiwan in 2002 and the number of single herbs or combined ingredients of Chinese herbal formulae.

There was an average of 4.8 Chinese herbs in a single prescription for subjects with insomnia. The most common number of herbal components in the prescribed Chinese herbal formulae or individual Chinese herbs for subjects with insomnia in Taiwan was six (20.0%), followed by five (17.3%) and three (15.8%; Figure 1). According to the association rule, the most commonly prescribed CHM drug combination for treating insomnia was Suan-zao-ren-tang with Long-dan-xie-gan-tang (Table 4), while the most common triple drug combination was Suan-zao-ren-tang, *A. julibrissin*, and *P. multiflorum* (Table 5). With regard to the most common quadruple drug combination, Suan-zao-ren-tang, Chai-hu-jia-long-gu-mu-li-tang, *P. multiflorum*, and *Poria cocos* accounted for 1.6% of all prescriptions, while the most common quintuple drug combination was Suan-zao-ren-tang, Gan-mai-da-zao-tang, *P. multiflorum, Poria cocos* and *Coptis chinensis*, which accounted for 1.6% of all prescriptions.

## 4. Discussion

The current study is the first large-scale survey of the use of CHMs for the treatment of insomnia in a Chinese population. This investigation was conducted by analyzing the computerized claim dataset of TCM office visits covered by the NHI in Taiwan. Our results showed that females were the more common TCM users (female : male = 1.94 : 1), which is in agreement with the results of a meta-analysis reported by Zhang and Wing [18]. We also found that nearly one half of the subjects (49%) that used TCM for insomnia were 30–49 years of age. Several factors, such as psychosocial problems, physical disorders or a patient's inclination toward TCM herbs, might account for the high prevalence rate of CHM use in this group [10, 19–21].

Clearly, a variety of CHM formulae or individual herbal drugs have been used to treat insomnia according to the practitioners' personal experiences or from the records of traditional Chinese texts. However, it remains unclear which of the Chinese herbal formulae or drugs used are the most effective in treating insomnia in clinical practice. Studies examining drug utilization and prescribing patterns through a large-scale survey of clinical practices can serve as an effective tool for investigating the clinical pharmacology of these compounds. In addition, these studies can provide relevant information for ways to screen and identify potentially effective CHM for treating insomnia [31, 32]. Once the effective Chinese herbs for treating insomnia are identified and confirmed in clinical trials, further research can be conducted in order to identify the bioactive ingredients of these herbs.

*Polygonum multiflorum* (Shou-wu-teng), the most commonly prescribed Chinese herb for subjects with insomnia, has been reported to have anti-inflammatory activity [33], an anti-atherosclerogenic effect [34] and exhibits neuroprotective effects in animal studies [35]. Shou-wu-teng is the climbing vine of *P. multiflorum*. According to TCM texts, its root can be used as medicine. However, the functions of the vine and root are different. Until the Qing Dynasty, the book “*Ben cao Zheng Yi*” of Chang Shan-Lei (1873–1934) indicated that Shou-wu-teng can treat insomnia. He stated that “there is a legend that the vine of this *P. multiflorum* will interconnect with each other at night, thus implying a sleep-promoting effect”. Although Shou-wu-teng is often used to treat insomnia during clinical practice, no clinical research exists in the Western literature verifying its sedative or anxiolytic effects. Importantly, there are some case reports demonstrating that Shou-wu-teng is associated with hepatotoxicity [36, 37]. Nevertheless, further clinical studies are needed to evaluate the efficacy and safety of Shou-wu-teng for treating insomnia in a clinical setting.

The second most commonly used Chinese herb for subjects with insomnia in our study was Suan-zao-ren (*Z. spinosa*). It is the chief ingredient in the formula of Suan-zao-ren-tang. In an animal model, Peng et al. [22] reported that Suan-zao-ren had a sedative effect at higher doses and an anxiolytic effect at lower doses. In addition, Zhang et al. [23] indicated that Jujuboside A, one of the components of Suan-zao-ren, produced its sedative-hypnotic effects through effecting the actions of anti-calcium-binding proteins and it inhibited the glutamate-mediated excitatory signaling pathway in the hippocampus. Jiang et al. [24] also reported that saponins, the main bioactive components of Suan-zao-ren, could prolong the sleeping time induced by barbiturates. In addition, Ma et al. [25] revealed that sanjoinine A, an alkaloid compound of Suan-zao-ren, might regulate GABAergic neurons and further increase the sleeping time and decrease the sleep latency induced by pentobarbital. Notably, there was a case report indicating that Suan-zao-ren could interact with the antidepressant, venlafaxine (Efexor), thereby leading to an acute serotonin reaction [38].

Other commonly prescribed individual Chinese herbs used for insomnia found in our study included the following: *Polygala tenuifolia* [26] and *S. miltiorrhiza* [27], which were reported to have sedative effects; and *A. julibrissin* [39], *Scutellaria baicalensis* [40, 41] and *Coptis chinensis* [42], which were reported to have anxiolytic effects in animal studies. Several individual Chinese herbs not included in our top 10 commonly used regimens are still used for the treatment of insomnia in clinical practice. Some of these Chinese herbs have been reported to have anxiolytic-like effects, including *Corydalis turtschaninovii* (Yan-hu-suo) [43], *Ginkgo biloba* (Bai-guo) [44], *Gardenia jasminoides* (Zhi-zi) [45], and *Gastrodia elata* (Tian-ma) [46]. Others have been reported to have hypnotic effects, such as *Stephania tetrandra* (Fang-ji) [28]. Certainly, these Chinese herbs are also worthy of further investigation regarding their clinical efficacy and safety in treating insomnia.

In the current study, Suan-zao-ren-tang was the most commonly prescribed Chinese herbal formula used for the treatment of insomnia. The use of Suan-zao-ren-tang was noted in the ancient Chinese book, *Synopsis of Prescriptions of the Golden Chamber*, written by Chang Chung Ching (AD 150–219), and has been used to treat insomnia for centuries. In the original article of this book, it stated the following [47]: “Consumptive disease with restlessness and insomnia can be treated with Decoction of Semen Ziziphi Spinosae”. In one clinical trial from Taiwan, Chen and Hsieh [11] concluded that Suan-zao-ren-tang improved the quality of sleep without generating significant side effects. In one survey from Hong Kong, Suan-zao-ren-tang was also the most common ingredient found in over-the-counter sleeping pills [48]. With regard to the basic researches examining the treatment of insomnia, current pharmacologic approaches have largely focused on the activity of gamma-aminobutyric acid A (GABA)_A_, one of the inhibitory neurotransmitters in the central nervous system [49]. In an experimental rat model, Suan-zao-ren-tang was shown to increase non-rapid eye movement sleep, and the mechanism was thought to be mediated through the stimulation of GABA_A_ and serotonin receptors [50, 51]. Importantly, Suan-zao-ren-tang is composed of five ingredients. Suan-zao-ren, the chief ingredient of Suan-zao-ren-tang, has been reported to have a sedative-hypnotic effect in animal studies [22–25]. Other ingredients of Suan-zao-ren-tang include *Anemarrhena asphodeloides*, *Poria cocos*, *Ligusticum chuanxiong* and *Glycyrrhiza uralensis (Radix glycyrrhizae)*. The latter herb has been shown to have an antidepressant-like effect through the inhibition of monoamine oxidase, leading to an increase in the levels of brain norepinephrine and dopamine in a murine model [52]. *P. cocos* extract was reported to enhance the secretion of cytokines, such as interleukin-1*β* and tumor necrotic factor (TNF)-*α*, in human peripheral blood monocytes [29]. In addition, these two cytokines have been shown to enhance non-rapid eye movement sleep [30]. In Table 6, we have summarized some important plants, which were prescribed, and clarify the major constituents and pharmacologic activities with sedative and hypnotic effects. Because Suan-zao-ren-tang is widely prescribed for the treatment of insomnia, it is necessary to conduct randomized, double-blind, placebo-controlled trials to assess its efficacy and safety in patients with well-defined sleep disorders.

Jia-wei-xiao-yao-san was the second most commonly used Chinese herbal formula for treating insomnia. According to one pilot clinical study [53], this formula can improve climacteric symptoms in postmenopausal women, such as anxiety, depression and insomnia. Other research has showed that Jia-wei-xiao-yao-san increased plasma levels of TNF-*α* in depressed menopausal patients [54]. Thus, this herbal formula might regulate cytokine levels in the central nervous system. In animal studies, Jia-wei-xiao-yao-san has been shown to have anxiolytic or antidepressent-like effects. Moreover, the underlying mechanisms were associated with the stimulation of the GABA_A_/benzodiazepine receptor and increased hippocampal neurogenesis [55, 56]. It is noteworthy that Kamisyoyo-san (a Japanese kampo formula or Jia-wei-xiao-yao-san in Chinese) was reported to induce adult respiratory distress syndrome after treating a case with seborrheic dermatitis [57].

Another popular Chinese herbal formula used in treating insomnia observed in our study was Chai-hu-jia-long-gu-mu-li-tang (Saiko-ka-ryukotsu-borei-to in Japanese). This formula can ameliorate sleep disorders by reducing excitation in an animal model [58]. In addition, Chai-hu-jia-long-gu-mu-li-tang has been reported to reduce stress-induced brain monoamine release, and may be used to treat depression [59, 60]. Gan-mai-da-zao-tang (Kanbaku-taiso-to in Japanese) has been shown to inhibit the hyperexcitability of neuronal membranes and may have sedative effects [61]. However, there are no reports regarding the use of Tian-wang-bu-xin-dan, Wen-dan-tang, Gui-pi-tang, Zhi-bai-di-huang-wan, Long-dan-xie-gan-tang (Yongdamsagan-Tang in Japanese) and Qing-xin-lian-zi-yin in the treatment of insomnia. In clinical practice, Long-dan-xie-gan-tang is commonly prescribed to subjects with chronic hepatitis in Taiwan [62]. Therefore, based on theories of TCM, it is possible that Long-dan-xie-gan-tang could treat insomnia manifested by the congestion of *qi* in the liver.

In general, practitioners of Western medicine attempt to determine the cause of insomnia and manage the patients accordingly [63]. In contrast, the principles of diagnosis and treatment of insomnia in Chinese medicine are primarily based on the manifestations of the syndrome (i.e., the chief signs and symptoms of patients), from which a corresponding prescription is provided [64]. The chief symptoms and signs according to the TCM diagnosis include appetite, thirst, mood, the color of the tongue and urine and the state of the pulse. The prescription is often a Chinese herbal formula. Moreover, one or more individual Chinese herbs can be added empirically to treat some of the other insomnia-related or non-related minor symptoms. In a typical Chinese herbal formula, the prescription is normally based on the concept of “emperor, minister, assistant, and servant” [65]. This concept emphasizes that a ministerial herb will have an additive or synergistic effect to the imperial herb (main ingredient), and an assistant herb can reduce the adverse effect of the imperial herb. Thus, we speculate that Chinese herbal formulae used for treating insomnia in this research present with their own corresponding TCM syndrome. For instance, Suan-zao-ren-tang is mainly used in treating insomnia with a syndrome due to internal heat and effulgent fire caused by liver blood deficiency. However, further investigation is warranted so as to confirm whether the corresponding TCM syndrome associated with Suan-zao-ren-tang means that this type of insomnia is the predominant syndrome in Taiwan or that the patients with this type of syndrome are in favor of TCM treatment.

In Taiwan, there are approximately 200 different Chinese herbal formulae and 300 individual Chinese herbs, which are available for use and are produced by TCM pharmaceutical companies that follow the regulation of good manufacturing practices [66]. In our study, there was an average of 4.8 components in each Chinese herbal formula or single-herb prescription for insomnia, perhaps representing the complexity of symptoms related to insomnia recognized by TCM physicians in Taiwan. According to our results, the most commonly prescribed CHM drug combination for treating insomnia was Suan-zao-ren-tang with Long-dan-xie-gan-tang, while the most common triple drug combination was Suan-zao-ren-tang, *A. julibrissin* and *P. multiflorum*. Although Suan-zao-ren-tang and Long-dan-xie-gan-tang are different for their syndrome differentiation, they are in the same prescription because it is difficult to clinically determine classical syndrome identification. However, we can also classify syndromes according to the cause of the disease, which is the so-called “disease cause syndrome identification”. For example, congestion of *qi* in the liver may cause liver blood deficiency. Thus, for syndrome differentiation and treatment, there is a cause and effect. We may also add a tranquillizing medicine according to the ancient medical textbooks. For example, *A. julibrissin* and *P. multiflorum* or other modern and pharmacologically-proven tranquillizers, may be added to this formula, that is, *Scutellaria baicalensis*. Nevertheless, further investigation is needed to examine if these herbal combinations have synergic effects or other beneficial effects in treating insomnia.

## 5. Conclusions

In conclusion, based on the availability of electronic healthcare claims data, a population-based pharmaco-epidemiology survey of Chinese herbs for treating insomnia was investigated. Thus, we now have a better understanding of the use frequencies and patterns of CHM prescriptions for the treatment of insomnia in a Chinese population. Nevertheless, the therapeutic effects and safety of these Chinese herbal formulae or individual herbs used in the treatment of insomnia requires further elucidation through efficiency-based clinical studies or well-designed randomized, double-blind, placebo-controlled trials.

## Figures and Tables

**Table 1:** Age-specific frequency for the use of Chinese herbal medicines in patients with insomnia under the National Health Insurance in Taiwan during 2002.

Age (years)	Subjects with insomnia using Chinese herbal medicines					
	Number of patients (%)		Male (%)		Female (%)	
0–9	65	(0.4%)	27	(0.17%)	38	(0.23%)
10–19	373	(2.3%)	151	(0.94%)	222	(1.38%)
20–29	2131	(13.2%)	677	(4.20%)	1454	(9.01%)
30–39	3839	(23.8%)	1273	(7.90%)	2566	(15.90%)
40–49	4074	(25.3%)	1381	(8.56%)	2693	(16.70%)
50–59	2743	(17.0%)	846	(5.24%)	1897	(11.76%)
60–69	1694	(10.5%)	596	(3.69%)	1098	(6.81%)
70–79	988	(6.1%)	447	(2.77%)	541	(3.35%)
≥80	227	(1.4%)	96	(0.60%)	131	(0.81%)

Total	16 134	(100%)	5494	(34.05%)	10 640	(65.95%)

Male : female = 1 : 1.94.

**Table 2:** The top 10 individual Chinese herbs prescribed for insomnia in Taiwan during 2002.

Chinese single herb (Chinese name)	Generic name	Number of prescriptions	Percentage
Shou-wu-teng	*P. multiflorum*	7093	23.8
Suan-zao-ren	*Z spinosa*	5459	18.3
Fu-shen	*Poria cocos*	3975	13.3
He-huan-pi	*A. julibrissin*	2982	10.0
Mu-li	*O. gigas*	2424	8.1
Yuan-zhi	*Polygala tenuifolia*	2373	8.0
Dan-shen	*S. miltiorrhiza*	2248	7.5
Huang-qin	*Scutellaria baicalensis*	2210	7.4
Huang-lian	*Coptis chinensis*	1595	5.4
Bai-he	*L. brownii*	1518	5.1

Total prescription numbers = 29 801.

**Table 3:** The top 10 Chinese herbal formulae prescribed for insomnia in Taiwan during 2002.

Chinese herbal formulae (Chinese name)	Ingredients	Number of prescriptions (%)
Suan-zao-ren-tang	*A. asphodeloides, G. uralensis (Gur), Ligusticum chuanxiong, P. cocos (Pco), Z. spinosa (Zsp)*	9299 (31.2%)
Jia-wei-xiao-yao-san (Dan-zhi-xiao-yao-san)	*Angelica sinensis (Asi), Atractylodes macrocephala (Ama), Bupleurum chinense (Bch), Gardenia jasminoides (Gja), Gur, Mentha haplocalyx, Paeonia lactiflora, Paeonia suffruticosa (Psu), Pco, Zingiber officinale (Zof)*	6303 (21.2%)
Tian-wang-bu-xin-dan	*Asi, Asparagus cochinchinensis, Codonopsis pilosula (Cpi), Ophiopogon japonicus (Oja), Platycladus orientalis, Platycodon grandiflorum, Polygala tenuifolia (Pte), Pco, Rehmannia glutinosa (Rgl), S. miltiorrhiza, Schisandra chinensis, Scrophularia ningpoensis, Zsp*	4656 (15.6%)
Chai-hu-jia-long-gu-mu-li-tang	*Bupleurum chinense, Cinnamomum cassia, Cpi, Os Draconis, O. gigas, Pinellia ternate (Pter), Pco, Rheum palmatum, Scutellaria baicalensis (Sba), Zof, Z. jujuba (Zju)*,	3704 (12.4%)
Wen-dan-tang	*Citrus aurantium, Gur, Phyliostachys nigra, Pter, Pco, Zof, Zju*	3445 (11.6%)
Gan-mai-da-zao-tang	*Gur, Triticum aestivum, Zju*	3427 (11.5%)
Gui-pi-tang	*Asi, Astragalus membranaceus (Ame), Ama, Aucklandia costus, Cpi, Dimocarpus longan, Gur, Pte, Pco, Zof, Zju, Zsp*	1960 (6.6%)
Zhi-bai-di-huang-wan	*Alisma plantago (Apl), Anemarhena asphodeloides, Cornus officinalis, Dioscorea opposite, Psu, Phellodendron chinense, Pco, Rgl*	1789 (6.0%)
Long-dan-xie-gan-tang	*Akebia trifoliate, Apl, Asi, Bch, Gja, Gentiana scabra, Gur, Plantago asiatica (Pas), Rgl, Sba*	1470 (4.9%)
Qing-xin-lian-zi-yin	*Ame, Gur, Lycium chinense, Nelumbo nucifera, Oja, Panax ginseng, Pas, Pco, Sba*	1370 (4.6%)

Total prescription numbers = 29 801.

**Table 4:** The most common prescription patterns for combination Chinese herbs in a single prescription for subjects with insomnia in Taiwan during 2002.

Chinese herbal formulae or single herbs	Support (%)	Number of prescriptions
Suan-zao-ren-tang, Long-dan-xie-gan-tang	2.0	597
Jia-wei-xiao-yao-san, Wen-dan-tang	2.0	593
Suan-zao-ren-tang, Zhi-bai-di-huang-wan	1.5	447
Wen-dan-tang, Chai-hu-jia-long-gu-mu-li-tang	1.4	422
Jia-wei-xiao-yao-san, Zhi-bai-di-huang-wan	1.3	379
Zhi-bai-di-huang-wan, *Z. spinosa*	1.2	364
Jia-wei-xiao-yao-san, *Corydalis turtschaninovii*	1.1	327
Suan-zao-ren-tang, Gui-pi-tang	1.1	319
Jia-wei-xiao-yao-san, Liu-wei-di-huang-wan	1.1	317

Total prescription numbers = 29 801.

**Table 5:** The most common prescription pattern for the triple drug combination of Chinese herbs in a single prescription for insomnia in Taiwan during 2002.

Chinese herbal formulae or single herbs	Support (%)	Number of prescriptions
Suan-zao-ren-tang, *A. julibrissin, P. multiflorum*	2.1	618
*Z. spinosa, P. multiflorum*, *A. julibrissin*	1.4	428
Tian-wang-bu-xin-dan, *P. multiflorum*, *A. julibrissin*	1.3	377
*L. brownie, P. multiflorum*, *A. julibrissin*	1.1	321
Jia-wei-xiao-yao-san, *P. multiflorum*, *A. julibrissin*	1.1	320
*P. cocos, P. multiflorum*, *A. julibrissin*	1.0	287
Suan-zao-ren-tang, Jia-wei-xiao-yao-san, *P. multiflorum*	0.9	279
Suan-zao-ren-tang, Jia-wei-xiao-yao-san, Tian-wang-bu-xin-dan	0.9	275
*Z. spinosa, Poria cocos, Polygala tenuifolia*	0.9	266
Suan-zao-ren-tang, *P. multiflorum, O. gigas*	0.9	256

Total prescription numbers = 29 801.

**Table 6:** Summary of major constituents and pharmacologic activities of Chinese single herbs prescribed for insomnia with sedative and hypnotic effects.

Chinese single herb (generic name)	Major constituent	Pharmacologic activity
*Z. spinosa*	Spinosin and jujubosides	Increases the hexobarbital-sleeping time and decreases the locomotor activity [22]
	Jujuboside A	Inhibits the glutamate-mediated excitatory signal pathway in the hippocampus [23]
	Saponins	Decreases monoaminergic system activity [24]
	Sanjoinine A	Regulates GABA-ergic systems and further increases the sleeping time and decreases the sleep latency induced by pentobarbital [25]
*Polygala tenuifolia*	3,4,5-Trimethoxycinnamic acid	Suppresses norepinephrine in the locus coeruleus of rats [26]
*Salvia miltiorrhiza*	Miltirone	Central benzodiazepine receptor partial agonist [27]
*Stephania tetrandria*	Tetrandrine	Effect on serotonergic system [28]
*Poria cocos*	Ergosterone	Enhances the secretion of the cytokines, interleukin-1*β* and TNF-*α*, which enhances non-rapid eye movement sleep [29, 30]
